#  Necessity of oral health intervention in schizophrenic patients - A review 

**DOI:** 10.3126/nje.v6i4.17254

**Published:** 2016-12-31

**Authors:** Swati Gupta, Pratibha PK, Richa Gupta

**Affiliations:** 1 Senior Research fellow, Department of Pharmaceutics and Nanotechnology, Panjab University (Chandigarh); 2 Head and Professor, Department of Periodontology, Manipal College of dental sciences (Manipal); 3 Assistant Professor, Department of Anatomy, Aadesh medical college (Haryana)

**Keywords:** Oral health; Schizophrenia, Treatment awareness.

## Abstract

Individuals with mental illness often cannot perform day to day activities due to a psychiatric or emotional disorder. Schizophrenia is one such psychiatric disorder characterized by worsening self-care ability with progressing mental illness. This disease may potentially deteriorate oral health by affecting the subject's ability to perform oral hygiene measures. Literature on oral disease manifestations in schizophrenia is limited. Lack of desire for oral health care as well as generally poor awareness of oral health issues in these patients, compounded further by side effects of medications, may complicate dental management in schizophrenic patients. The present review explores clinical features and possible factors associated with oral health status among those with Schizophrenia.

## Introduction

Diseases affecting the oral cavity are frequently neglected or under diagnosed; having a great impact on an individual's health in the long term [  1 ]. Therefore, maintenance of oral health becomes imperative for one's general wellbeing. Moreover, a healthy dentition is increasingly being perceived as important for self-image and confidence in affairs of routine life [  2 ]. 

 Oro systemic health interrelationship becomes ever more relevant in cases of mental illness. Oral health care is of particular concern in mentally challenged and institutionalized patients, as poor oral hygiene not only socially excludes these patients but also predisposes them to various other systemic health problems like respiratory infections and cardiac diseases. Insufficient institutionalized care by caretakers augments the problem. Measures to institute plaque control in conditions such as Down's syndrome / retardation in children are still manageable to an extent through concerted efforts on the part of the parents, nurses and dental professionals. However, certain psychiatric disorders such as schizophrenia warrant special attention as the health condition is relatively difficult to identify, diagnose and manage; compounding the dental problems further. 

 Disturbances in thoughts, behavioral changes, impaired cognitive functions which affect a person's ability to work or communicate socially and incapability of self-care characterize schizophrenia [  3 ].It is often associated with positive and negative symptoms. Hallucinations, conversing voices with or about the patient, and paranoid delusions are few of the positive symptoms whereas 'negative' symptoms are flattened affect, loss of a sense of pleasure leading to social withdrawal, isolating the patient who eventually loses the will or drive to perform routine activities [  4 ]. Presently, the diagnostic criteria put forward by the World Health Organization and the American Psychiatric Association are widely used for the diagnosis of schizophrenia. Few signs and symptoms considered essential for confirming the diagnosis include symptom duration of 6 months, delusions, hallucinations, disorganized speech or behavior, affective flattening, alogia, and avolition, social and occupational dysfunctions [  5 ]. These features make it difficult for the affected individual to carry out daily activities and maintain personal hygiene. The incidence rate of schizophrenia has been reported to be 7 per thousand of the adult population, mostly in the age group of 15-35 years. Due to the chronic nature of the condition, it has a high prevalence of about 24 million people worldwide [  6 ]. An estimated 85 percent of older adults with schizophrenia develop their illness before age of 45 years, mostly in their second or third decade of life [  7 ].

Genetics is considered to be a major reason, amongst many etiological factors causing schizophrenia. Rare genetic mutations and other early influences, such as gestational and birth complications are also thought to be involved in the causal pathways for schizophrenia [ 8 ]. Changes in levels of neurotransmitters, dopamine and serotonin, in the brain has been suggested to be a probable mechanism for this condition [ 9 ]. Social isolation, hostile family atmosphere and drug abuse may be other reasons predisposing to this condition [ 10 , 11 ]. 

 Poor oral health status in schizophrenics has been attributed to factors like poverty, less resources, imbalance of mental status, medications and most of all the effect of systemic diseases like diabetes, hypertension, osteoporosis which reduce the self-care ability of the patients [ 12 ]. A study in institutionalized patients found the oral hygiene status to be poorer in schizophrenic patients [ 13 ]. It has also been suggested that oral health deteriorates with increasing age, length of hospitalization and has been found to be worse amongst inpatients as compared to outpatients [ 14 ]. 

 Higher rates of caries and multiple missing teeth compared to healthy subjects, has been reported in patients with mental illness [ 15 ]. A survey was conducted on 565 institutionalized patients, out of which 62% were diagnosed with schizophrenia. Higher DMFT scores were observed in female patients and those with dementia. It was concluded from the survey that decayed-missing-filled teeth (DMFT) score increased with age [ 16 ]. Socioeconomic status is another factor that poses a risk for schizophrenia development. Demanding household and harsh community environments may be responsible for poor access to social services like health care and education, often leading them to social exclusion [ 17 ]. Hence, those in the lower socio economic classes may show many more teeth extracted, decayed, and filled, than those from a higher class [ 18 ]. 

 These patients generally are prone to adverse orofacial effects such as xerostomia, sialorrhea and oral dyskinesia caused by psychotropic medications [ 19, 3 ,20 ]. The first generation anti-psychotics (haloperidol, chlorpromazine, thioridazine, trifluoperazine), used for schizophrenia treatment, are known to cause hypo-salivation by blocking the parasympathetic stimulation of the salivary glands, putting the patients at a high caries risk, particularly root caries [ 21 ]. Due to xerostomia various other lesions like candidiasis, glossitis, generalized stomatitis set in, affecting speech and swallowing or leading to poor denture tolerance, instability of dentures or denture trauma. Dyskinesia and dystonia seen in these patients affect motor activities producing abnormal jaw movements, giving rise to presentations such as tongue protrusion, retraction and facial grimacing [ 22 ].

Fear and anxiety in these patients may manifest as premature tooth wear [ 23 ]. Most of the schizophrenic patients have some kind of Para functional habit like nail biting, lip biting etc. Bizarre behaviour resulting in self-mutilation in the form of autoextractions [ 24 ], glossectomy [ 25 ] and excoriation of gingival tissues with sharp fingernails [ 26 ] has also been reported. 

 Over 60% of schizophrenics are found to be heavy smokers [ 27 ]. In an institutional study, the smoking prevalence was more in psychiatric outpatients as compared to local or national population-based samples (52% versus 30% and 33%). Factors like age, sex, marital status, socioeconomic status, alcohol use, coffee use, or institutionalization of the psychiatric patients had no bearing on the increased prevalence rate. Schizophrenics (88%), Maniacs (70%) and severely ill patients were found to be the heavy abusers [ 28 ]. The reasons for such high frequency of heavy smoking prevalent in schizophrenic patients is thought to be partially related to its enhanced stimulant effect. Smoking induces release of dopamine in the brain, stimulating its activity and inhibiting its degradation [ 29 ]. 

 Cigarette smoking in these patients may predispose them to periodontal disease and oral cancer. A cross-sectional survey on 250 schizophrenic patients investigated the relationship between periodontal disease and schizophrenia. An increased gingival and plaque index, higher probing depths in chronic schizophrenic patients (P < 0.001) pointed towards poor periodontal conditions in these patients [ 30 ]. Smoking is one of the contributory factors to poor periodontal health, which is characterized by increased pocket depth, periodontal attachment loss leading to increased incidence of tooth loss [ 29 ]. The prevalence of salivary P. gingivalis in schizophrenic patients has been reported to many folds higher when compared to non-psychiatric controls. In one study, a quantitative relation was established between severity of schizophrenia and P. gingivalis cells [ 31 ]. 

 The chronicity of the illness has been attributed to negative symptoms, which are potentially devastating to oral health as they impair a patient's desire to maintain a good oral hygiene.14 Poor self-care, neglected oral care, low perception of dental treatment needs, and poor diet may further contribute to oral neglect in such patients. Therefore routine dental care becomes a challenging task for the patient, caretaker, and the physician. Tremors have been observed as side effects of antipsychotic medications [ 32 ]. A cross sectional study correlated the association between tremors and poorer dental status. Tremors interfere with day to day fine motor skills of the patient, impairing one's tooth brushing, hence again leading to poor oral health [ 33 ].

The reasons for infrequent dental visits in schizophrenics and patients with mental illness have been observed as lack of drive to maintain self-care, due to poor general health, inability to meet cost of treatment and low priority for dental check-up, unless, in case of emergency [ 34, 35 ]. Dental care associated behavior assessment in 372 psychiatric inpatients showed that these patients have less impetus to visit a dentist. They brushed their teeth for shorter periods and did not realize that oral health may have an influence on their general health condition [ 34 ]. 

 Attitude of dental professionals and limited knowledge of causes and effects of oral diseases in this special group of patients, are other issues resulting in delay in providing treatment. Instances of low tolerance on the part of the dental staff in dealing with patients with low compliance, and unwillingness to treat such patients has also been reported [ 36 ]. Due consideration to these factors by dental professionals and modification of treatment plans to specifically cater to these patients will motivate them to accept dental treatment and also reduce the number of dental visits [ 37 ]. 

 While managing patients with this chronic illness, a dentist should take utmost care of the oral hygiene maintenance. A thorough history regarding the patient's oral hygiene practices, tobacco consumption, parafunctional and other abusive habits, medications as well as attitude of the patient's family towards oral health should be taken into consideration. The patient should be dealt with empathetically and treated like any other patient. Good communication and rapport should be built to avoid any discomfort to the patient. Priority should be given to counseling of the care takers, oral prophylaxis, restoration of decayed teeth, oral rehabilitation of edentulous patients and any emergency treatment possible. 

 Patient's physician should be consulted for recall and further maintenance of the patient's oral health. Oral health education programs can be organized for institutionalized patients to interact with patients and educate them about good oral hygiene practices. This would also meet their dental care needs and provide easy access to these patients. The effective use of tooth brushes, non-alcohol mouth washes can be introduced in the daily regime of the patient with the coordination of the patient's care taker. Moreover, dentists can take up a more active role in helping these patients with smoking cessation [ 38 ]. 

 In patients with periodontal problems, routine oral prophylaxis can be performed. Surgical procedures like root planing and flap surgeries are not a contraindication and can be performed with precautions in patients who are stable and under medication. Health care professionals should be trained and updated regarding the oral diseases, side effects of medications, and possibility of dental treatment for these chronically ill patients. Schizophrenic patients should not be refused treatment on grounds of inability to maintain oral hygiene.

Hence, it becomes imperative on the part of the patient's family and other health care professionals to assimilate knowledge on this medical condition and its management and also keep themselves abreast with newer advances in the field. The traditional view of assuming oral health care as secondary to other aspects of systemic health must give way to the positive impact of improved oral health care on general health. 

 To conclude, some of the clinical features of this illness are not evident and therefore the dental practitioner must understand the more common presentations and management considering that poor dental condition in schizophrenics may be associated with an increased risk of systemic comorbidity as well. Physicians have to work in coordination with dental practitioners to maintain good oral health. Therefore, knowledge and awareness of different aspects of this disease will help dental professionals in diagnosis and planning of better treatment strategies for this specific population.

## Figures and Tables

**Table 1.** Table 1: : Oral health status in schizophrenics

**Authors **	**Study design**	**Parameters recorded **	**Sample size**	**Results**	**Conclusion**
**EltasA** **, ** **KartalcS, EltasSD, Dundar S, Uslu MO** **(2013)** (40)	Cross-sectional study Group A- medications causing xerostomia Group B- medications causing sialorrhea	Plaque index (PI), Bleeding on probing (BoP), Probing pocket depth (PPD) and Clinical attachment levels (CAL), DMFT	Group A-20 Group B- 20	PI and BOP higher in group A PPD, CAL, DMFT similar in both groups	Higher risk of periodontal disease in patients with schizophrenia irrespective of the medication.
**Persson K** **, ** **Axtelius B, Soderfeldt B** **, ** **Ostman M** **(2009)**	Descriptive Population surveys of outpatient under psychiatric care.	DMFT and oral hygiene correlated with age and no. of antipsychotic drugs	113	Oral health neglected in men more than women	Regular dental check-ups should be encouraged for patients under psychiatric care
**ArnaizA** **, ** **ZumarragaM, Díez-Altuna I** **, ** **Uriarte JJ, Moro J** **, Perez-Ansorena MA** **(2011)**	Evaluate the oral health of a group of schizophrenic outpatients and a control group without psychiatric illness.	DMFT, CPITN, PANSS index,		DM index, CPITN higher in schizophrenics. Filled teeth were lower than controls. PANSS negative score correlated with oral health variables	Oral health in psychiatric patients is poorer than others irrespective of habits and age.
**Chu KY, Yang NP, Chou P, Chiu, Hsien J, Chi LY (2010)** (14)	Cross-sectional survey	DMFT, community periodontal index in schizophrenic patients	1103	DMFT index was 14.3%; 5% were edentulous, 39.4% had (community periodontal index ⩾ 3) Aging men with lower educational levels and a longer stay in institutions were likely to have lower filling rate of the DMFT index	Long-term care institutions that care for inpatients with schizophrenia should exert greater efforts in providing dental care
**Tani H, Uchida H, Suzuki T, Shibuya Y, Shimanuki H, Watanabe K** **(2012)** **(35)**	Cross-Sectional study	DMFT score: age, sex, smoking status, daily intake of sweets, dry mouth, frequency of daily tooth brushing, tremor,	523 patients in age group of 40-60 years	Univariate general linear model older age, smoking, tremor burden, and less frequent tooth brushing were associated with a greater DMFT score.	Schizophrenia patients who do not regularly brush their teeth or who exhibit tremor, it may be advisable for caregivers to encourage and help them to perform tooth brushing more frequently.
**McCreadie RG** **, Stevens H** **, Henderson J** **, Hall D** **, ** **McCaul R, Filik R (2004)** (21) ****	Descriptive study	Self-report questionnaire	428 people with schizophrenia in six different areas of the UK	More of the younger patients were edentate (3-39% vs. 1-20%) and fewer had more than 20 teeth (70% vs. 83%). Fewer patients cleaned their teeth daily; this group had more negative symptoms.	Dental health of people with schizophrenia is poor. Community mental health teams should encourage them to attend their community dentist regularly.
